# Adequacy of energy and macronutrients intake in differently active slovenian adolescents

**DOI:** 10.1186/s40795-023-00708-x

**Published:** 2023-03-27

**Authors:** Emanuela Čerček Vilhar, Petra Golja, Gregor Starc, Barbara Koroušić Seljak, Katja Zdešar Kotnik

**Affiliations:** 1 Biotechnical Faculty, Department of Biology, University of Ljubljana, Vecna pot 111, Ljubljana, 1000 Slovenia; 2 Faculty of Sport, University of Ljubljana, Gortanova 22, Ljubljana, 1000 Slovenia; 3 Computer Systems Department, Jožef Stefan Institute, Ljubljana, 1000 Slovenia

**Keywords:** Energy intake, Macronutrient intake, Physical activity, Adolescents

## Abstract

**Objective:**

Evaluate the adequacy of energy/macronutrient intake in adolescents according to the Slovenian national recommendations adopted from the recommendations of the German Nutrition Society and to identify differences in energy/macronutrient intake between differently active adolescents.

**Methods:**

Data on energy and macronutrient intake (24-hour dietary recall), physical activity (SHAPES questionnaire), and anthropometric characteristics (body mass and height) of adolescents were obtained on a representative sample of first-year secondary school students (average (SD) age: 15.3 (0.5) years; N = 341), who were included in the national survey The Analysis of Children’s Development in Slovenia (ACDSi) in 2013/14.

**Results:**

75% of adolescents met the national recommendations for carbohydrates and proteins and 44% for fats, whereas only 10% of adolescents met the recommendations for energy intake. Energy/macronutrient intakes were significantly higher in vigorously physically active (VPA) boys compared to moderately (MPA) and less (LPA) physically active boys. No such differences were observed between girls of different physical activity levels.

**Conclusion:**

Adolescents need to be encouraged to meet their energy needs according to gender and physical activity (especially VPA girls) and to reach for higher quality foods in adequate macronutrient proportions.

## Introduction

Adolescence is one of the most intense periods of growth and development in human life, during which major physical and hormonal changes occur [1]. Consequently, an increased energy and macro- and micronutrients intake is required [1]. In order to maintain existing body mass, energy balance must be maintained, which is achieved when a person’s total daily energy intake equals to total daily energy expenditure [2]. Inadequate dietary intake during adolescence can lead to delayed growth, endocrine dysfunction, impaired cognitive function, sexual development disorders, and bone mass loading disorders [2], [3]. In contrast, excessive dietary intake and physical inactivity can lead to a positive energy balance and the development of overweightness and obesity [4]. Although nutritional deficiency may seem paradoxical in terms of excessive food intake, diet may include micronutrients-poor food that lacks required amounts of micronutrients and therefore overweight and obese individuals may suffer from vitamin and mineral deficiencies. Indeed, several studies have already revealed the association between obesity and micronutrient deficiencies, which have been documented for iron [5], vitamin D [6], and zinc [7] deficiency. Obesity in children and adolescents can lead to both, short term consequences such as mental (i.e. low self-esteem, eating disorders, attention deficit hyperactivity disorder, depression), and physical (i.e. high blood pressure, dyslipidaemia, hyperinsulin aemia and/or insulin resistance, asthma, type 1 diabetes, chronic inflammation) health problems, as well as long term adverse consequences on physical morbidity (cardiometabolic diseases and cancer) and thus premature mortality in adulthood [8], [9], [10], [11].

Due to rapid physical changes during adolescence, adolescents may have difficulty accepting their bodies, and they are also more susceptible to the influences of the media and their peers than adults. These are all factors that can negatively affect adolescents’ eating habits [12]. They start skipping breakfast, while snacking, eating at restaurants and fast food chains, and consuming sweet and high-energy drinks remain common [12], [13]. Reports indicate that adolescents generally consume large amounts of total and saturated fats, salt and sugar, but too little complex carbohydrates and dietary fiber [3]. Of particular concern is the fact that the majority of obese adolescents remain obese into adulthood [14].

A key role in maintaining adequate energy consumption is physical activity, which has unfortunately been decreasing in adolescents in recent years [15]. Furthermore, studies have revealed that adolescents spend growing amounts of their free time in front of screens, which results in insufficient amount of sleep, which can contribute to the development of overeating behaviors and eventually to obesity [16]. In contrast, although less frequent, malnutrition has also been observed in adolescents due to inadequate energy intake over a long period of time [17]. Studies have shown that inadequate energy and nutrient intake due to increased physical activity is most prevalent in young athletes and physically more active individuals. Among athletes, inadequate nutrient intake can increase the incidence of sports injuries, negatively affect recovery, motor performance, and sports results [18], and long-term deficiency can lead to the development of Relative energy deficiency syndrome in sport (RED-S) among athletes [19], [20].

Maintaining an adequate energy and macronutrient balance is therefore crucial for normal growth and development of adolescents and for preventing long-term health consequences. Adolescents can maintain their energy balance primarily through healthy, balanced diet and adequate extent of physical activity [2]. Thus, the aim of our study was to determine the adequacy of energy and macronutrients intake (carbohydrates, proteins, and fats) in Slovenian adolescents, relative to their daily physical activity. We expected this would allow us to identify vulnerable subgroups of adolescents in terms of nutritional intake.

## Methods

### Study design

The present study is part of the larger cross-sectional study The Analysis of Children’s Development in Slovenia (ACDSi) 2014, conducted every ten years in Slovenia on a representative sample of children and adolescents [21]. The protocol of the study was approved by the National Medical Ethics Committee of the Republic of Slovenia (52/03/14 and 66/11/12) [21].

### Study sample

The sample was selected in a two-stage procedure with clustered and stratified sampling. In the first stage, 16 out of 170 Slovenian secondary schools were selected based on the secondary school educational programme and geographical location. In order to ensure the national representativeness of the sample not only by gender, age (about 200 males and 200 females from each of the four years of secondary school education), and geographical location, but also by secondary school programme, the ratio of sampled students from different secondary school educational programmes was maintained equal to the ratio at the national level in Slovenia (data from the Statistical Office of the Republic of Slovenia). In the second stage of sampling, a required number of classes from each school were randomly selected for participation. If the number of students from the selected classes was insufficient due to lack of their responses or other reasons, additional students from other classes in the same school were randomly included in the sample. The final ACDSi survey sample included participants who were enrolled in 15 public high schools, provided informed consent signed by their parents, and completed the survey (n = 1479). The ACDSi sample represented 5% of the total national high school population and was ensured to be representative in terms of geographic location, diversity of national school programs, gender, and age.

The students, who were enrolled in the first-year of secondary school educational program, were presented with an additional section on nutrition in the questionnaire. Therefore, this subsample was included in the analysis of the present study. The inclusion criteria for the final analysis was that the participants had to participate in both 24-h recalls (n = 341).

### Data collection

#### Assessment of dietary intake

Daily energy, carbohydrate, protein, and fat intakes of participating adolescents were estimated using a 24-hour recall method following the recommendations of the European Food Safety Authority (EFSA). In each school, trained interviewers conducted a 24-hour recall computer-assisted interview. In accordance with EFSA standards, the method was performed twice with the same participant, two weeks to one month apart. Interviews at each school were conducted from Monday to Friday, so food intake of a weekend day was recorded for all participants who performed interviews on Mondays. During the interview, participants were asked to indicate exactly what foods they had consumed at all meals, including all snacks and beverages, the day before the interview. To estimate the amount of food consumed, a picture book of portion sizes [22] and national household measures (e.g., 1 tablespoon of honey, 1 cup of milk, 1 glass of water, 1 piece of bread, etc.) were used. All collected data were carefully reviewed by the trained interviewer (12 different interviewers conducted 24-recall on-site) and, if necessary, additional information on food intake was obtained from the adolescents. Subsequently, the interviewer entered all data into the Slovenian web-based tool for dietary assessment named Open Platform for Clinical Nutrition [23], which was used to obtain data on the energy and macronutrient composition of foods and beverages. All the entries were double checked by 2 trained interviewers. If the foods and recipes entered into OPEN did not yield a specific energy value and/or lacked data for macronutrients, the entries were replaced with similar foods and/or recipes for which the corresponding values were available. In turn, we considered the average dietary intakes calculated by the OPEN tool from the two 24-hour recalls, which was only feasible for those individuals who completed both 24-hour recalls. The energy and micronutrient data were then exported from OPEN to the MS Excel spreadsheet for each individual for further analysis. The obtained data on estimated daily intake of carbohydrates, fats, proteins, and energy were compared with the Slovenian national reference values [24], separately for boys and girls. The same reference values were used for all adolescents in our sample: those for the age group of 15–18 years.

#### Assessment of physical activity

Physical activity was assessed with a questionnaire School Health Action, Planning and Evaluation System (SHAPES) [25] questionnaire, that was originally developed for the Canadian population. For the ACDSi survey [21], it was translated into Slovenian and converted into an electronic form. Prior to the beginning our measurements, we tested it for reliability and validity at one of the schools that were later on not included in the sample, and established that it was suitable for the needs of our study. Originally, the questionnaire SHAPES contain 45 questions, but in the present study only questions on physical activity were used. When completing the questionnaire, participants reported the amount of time they were vigorously (VPA) and moderately (MPA) physically active in the last seven days. From the MPA and VPA data obtained, average daily energy expenditure for physical activity (DEEPA; kcal/kg·day) was calculated using the method of Wong and Leatherdale [26], separately for each participant. Shortly, based on the data on VPA and MPA, we calculated the average daily physical activity energy expenditure (DEEPA) for each subject (calculated separately for VPA and MPA). For this purpose, the total weekly amount of physical activity was first calculated by adding the number of hours a subject spent in physical activity on seven days separately for VPA and MPA. Then the sum for VPA or MPA was divided by seven (i.e., by the number of days in the week) to obtain the average amount of VPA or MPA expressed in hours/day. DEEPA was then expressed in kilocalories per kilogram of body mass per day (kcal/kg·d) using the method of Wong and Leatherdele [26], where the average daily duration of VPA (expressed in hours) was multiplied by 6 metabolic equivalents (MET) and the average daily duration of MPA (expressed in hours) was multiplied by 3 MET, according to the equation: average DEEPA = (hours spent daily with VPA x 6MET) + (hours spent daily with MPA x 3 MET); where 1 MET = 1 kcal / kg·hour. Adolescents were then classified into three categories, based on the calculated value of DEEPA: into less active (DEEPA below the 16th percentile), moderately active (DEEPA between the 16th and 84th percentiles), and vigorously active individuals (DEEPA above the 84th percentile) [26].

#### Anthropometric measurements and body mass index (BMI)

Body mass and body height data were obtained with anthropometric measurements. Measurements were performed according to standard protocols described by Lohman et al. [27] using SECA 769 scale (Seca Gmbh & co., Hamburg, Germany) and anthropometer GPM 101 (Siber & Hegner, Zürich, Switzerland). BMI was subsequently calculated as the ratio of body mass (kg) per square of body height (m^2^). Subjects were classified into categories of normal weight, overweight, obese, and underweight using the cut-off points proposed by Cole et al. [28], [29].

### Data analysis

Normality of the data distribution was tested using the Shapiro-Wilk test. Because of the asymmetric distribution (food intake, age…), non-parametric tests were used to test for statistical differences, and data were presented as median values and 5th and 95th percentile values. The reference values for energy intake from the national recommendations (the Slovenian national recommendation was adopted by the Nutrition Societies of Germany, Austria, and Switzerland) are estimated based on physical activity level (low, moderate, and high) [24], so we used the DEEPA calculation to classify participants into these three groups. Mann-Whitney U-test (for two independent samples) was used for: (a) gender differences in body mass index (BMI; kg/m^2^), average DEEPA, energy, and macronutrient intake (protein, carbohydrate, and fat); (b) gender differences in energy and macronutrient intake (protein, carbohydrate, and fat) in groups of adolescents with different DEEPA; (c) comparison of BMI and DEEPA between adolescents who met and did not meet energy intake recommendations; (d) the assessment of gender differences in compliance with the existing recommendations for energy and macronutrients intake, for all adolescents combined and also for differently active adolescents.

The nonparametric Kruskal-Wallis test was performed to compare energy and macronutrient intakes between differently physically active participants (i.e., less / moderately / vigorously physically active; LPA, MPA, and VPA). A post-hoc test to assess differences between the groups was performed where Kruskal-Wallis test showed statistically significant differences.

The Pearson’s chi-square test of independence for 3 × 2 contingency tables was used to compare percentages of differently active adolescents who met or did not met Slovenian national reference values (RV) for daily intake recommendations for energy and macronutrients. The p value was adjusted to 0.008 with the Bonferroni correction due to multiple comparisons.

All data were analyzed using IBM SPSS Statistics 22 (IBM, Armonk, New York, USA). The level of statistical significance was set at 0.05.

## Results

Our sample included 341 adolescents (girls n = 179, 52.5%; boys n = 162, 47.5%) from the first year of secondary school. The average (± standard deviation) age of the adolescents was 15.3 (± 0.5) years (range 14 to 18 years).

Boys were on average taller and heavier than girls. Height was 174.9 (± 7.0) and 165.3 (± 6.5) cm, respectively (p < 0.001). Body mass was 65.5 (± 10.9) and 59.3 (± 10.9) kg, respectively (p < 0.001). According to BMI, 75.4% of adolescents were of normal weight (average BMI was 21.1), 16.9% were overweight (average BMI was 26.7), 3.0% were obese (average BMI was 34.0), and 4.7% were underweight (average BMI was 17.6). Results demonstrated that 1.8% of adolescents were not physically active at all, while the most physically active adolescent had an average DEEPA (based on self-reported values) of 30.3 kJ/kg·day. The calculated median (5. – 95. percentile) values for average DEEPA was 2.3 (0.0 -3.4) in LPA adolescents, 7.6 (3.9–12.9) in MPA adolescents, and 9.5 (13.3–23.6) in VPA adolescents. Boys were significantly more physically active than girls (p < 0.001).

### Absolute dietary intake in adolescents

The calculated median intake values for energy and macronutrients, physical activity extent, and BMI are presented in Table 1 relative to gender. A comparison between genders demonstrated that girls consumed significantly less energy per day than boys (p < 0.001). The intake of all macronutrients (i.e. carbohydrates, proteins, and fats) was also significantly higher in boys than in girls (p < 0.001). In general, higher extent of physical activity was observed in boys than in girls, however, the result was statistically significant only in the MPA group of adolescents (p = 0.002). There were no statistical differences between boys and girls relative to BMI in any of differently physically active groups.

**Table 1:** Average daily energy intake, macronutrient intake, physical activity extent, and body mass index in adolescents

	All n = 341		Boys n = 162		Girls n = 179		
	**M**	**5. − 95. percentile**	**M**	**5. − 95. percentile**	**M**	**5. − 95. percentile**	**p**
Energy (kcal)	1733	937–3161	2112	1179–3562	1481	865–2541	0.000
Energy (kJ)	7256	3923–13,234	8843	4936–14,913	6201	3622–10,639	0.000
Carbohydrates (g)	236	123–434	275	154–481	207	104–353	0.000
Carbohydrates (%)	54	43–65	54	43–65	56	45–65	0.001
Proteins (g)	68	31–134	84	46–150	54	28–91	0.000
Fats (g)	54	25–118	69	31–135	49	22–91	0.000
Fats (%)	29	20–39	29	20–40	29	19–39	0.30
DEEPA (kcal/kg·day)	7.7	1.6–1.3	9.5	2.2–18.0	6.2	1.3–16.3	0.000
DEEPA LPA (n = 55)	2.3	0.0–3.4	2.3	0.0–3.4	2.3	0.1–3.3	0.50
DEEPA MPA (n = 224)	7.6	3.9–12.9	8.9	3.9–13.0	6.8	3.8–12.6	0.002
DEEPA VPA (n = 56)	9.5	13.3–23.6	15.4	13.3–26.1	16.4	14.4–21.3	0.13
BMI (kg/m^2^)	20.7	17.3–27.7	20.7	17.3–26.7	20.8	17.3–28.4	0.55
underweight (n = 13)	16.9	14.8 - NA	16.8	16.8 - NA	16.8	14.8 - NA	0.41
normal weight (n = 259)	20.3	17.8–23.4	20.2	17.6–23.1	20.4	17.8–23.6	0.31
overweight (n = 55)	25.6	23.6–28.4	24.7	23.4–27.4	26.1	23.7–28.5	0.03
obese (n = 11)	31.6	28.4 - NA	33.2	31.6 - NA	30.8	28.4 - NA	0.32

n, number of participants; M, median; NA, not available due to small sample; g, gram; kcal, kilocalorie; kJ, kilojoule; DEEPA, average daily energy expenditure for physical activity; LPA, less physically active; MPA, moderately physically active; VPA, vigorously physically active; BMI, body mass index; significance results obtained with Mann-Whitney U-test for two independent samples between boys and girls are presented with exact p-values

As demonstrated in Table 2, the differently active boys had significantly different intake of energy (p = 0.02), carbohydrates (p = 0.03), proteins (p = 0.002), and fats (p = 0.02)). A more detailed post-hoc analysis demonstrated that VPA boys consumed significantly more energy compared to MPA boys (p = 0.01). There was no significant difference in energy intake between LPA and VPA boys (p = 0.21) and between LPA and MPA boys (p = 1.0). The same was true for carbohydrate intake (p = 0.03), although a larger intake was observed (p = 0.02) in MPA than in VPA boys. Differences between groups of differently physically active boys were also found in protein intake, namely, VPA boys consumed more proteins than LPA boys (p = 0.005), as well as MPA boys (p = 0.01). VPA boys consumed more fat than MPA boys (p = 0.02), while there was no significant difference in fat intake between LPA and VPA boys (p = 0.91) and MPA and LPA boys (p = 1.0). In girls, no statistically significant differences were found in energy (p = 0.91), carbohydrate (p = 0.83), protein (p = 0.96), or fat (p = 0.90) intake between girls with different physical activity extent. Additional gender comparisons revealed that the energy and all macronutrients (i.e. carbohydrates, proteins, and fats) intake was significantly higher in boys than in girls in all groups of physical activity (p < 0.005). Interestingly, there were no statistically significant differences in BMI between differently active adolescents.

**Table 2:** Body mass index, energy, and macronutrients intake in differently physically active adolescents. n, number of subjects; LPA, less physically active; MPA, moderately physically active; VPA, vigorously physically active adolescents; BMI, body mass index; M, median; significance results obtained with Kruskal-Wallis test between differently physically active adolescents are presented with exact p-values

	BMI (kg/m^2^)			Energy (kcal)			Carbohydrates (g)			Proteins (g)			Fats (g)		
	**M**	**5. − 95. percentile**	**p**	**M**	**5. − 95. percentile**	**p**	**M**	**5. − 95. percentile**	**p**	**M**	**5. − 95. percentile**	**p**	**M**	**5. − 95. percentile**	**p**
**All; n = 338**															
LPA (n = 55)	20.5	17.0–35.0	0.12	1654	1007–3017	0.000	236	123–370	0.002	60	34–96	0.000	53	29–109	0.01
MPA (n = 224)	20.9	17.5–27.6		1686	909–3022		230	121–421		67	31–127		53	25–109	
VPA (n = 56)	20.5	17.2–26.9		2110	1023–4142		277	122–553		83	23–165		69	24–142	
**Boys; n = 162**															
LPA (n = 19)	20.4	16.8–24.6	0.40	2105	1111–3169	0.02	276	137–408	0.03	73	46–98	0.002	70	312 − 114	0.02
MPA (n = 100)	21.0	17.3–26.7		2052	1117–3222		262	140–476		81	45–135		66	30–127	
VPA (n = 40)	20.5	17.3–27.0		2382	1332–4517		304	196–633		91	41–183		83	35–151	
**Girls; n = 176**															
LPA (n = 36)	20.8	15.9–38.5	0.47	1498	936–2468	0.91	212	104–352	0.83	55	27–91	0.96	47	29–86	0.90
MPA (n = 124)	20.9	17.4–28.3		1470	873–2588		204	112–347		55	28–93		49	22–92	
VPA (n = 16)	20.4	15.9–25.3		1589	638–2804		214	89–376		55	19–93		51	22–105	

n, number of subjects; LPA, less physically active; MPA, moderately physically active; VPA, vigorously physically active adolescents; BMI, body mass index; M, median; significance results obtained with Kruskal-Wallis test between differently physically active adolescents are presented with exact p-values

### Meeting national recommendations for dietary intake in adolescents

A comparison of adolescent energy and macronutrient intake with national recommendations [24] is presented as the percentage of adolescents who met / did not meet daily recommended value for energy and all macronutrients (carbohydrates, proteins, and fats) according to gender (Table 3) and physical activity (Table 4).

**Table 3:** Percentage of adolescents, who met/did not meet reference values (RV) for daily energy and nutrient intake

	Boys (n = 162)					Girls (n = 179)*							
	**Met** **RV**		**Did not** **meet RV**			**Met** **RV**		**Did not** **meet RV**					p
	n	%	n	%		n	%	n	%				
**Energy**	20	12.3	142	87.7		15	8.5	161	91.5			0.17	
**Carbohydrates**	114	70.4	48	29.6		142	79.3	37	20.7			0.06	
**Proteins**	134	82.7	28	17.3		120	67.0	59	33.0			0.001	
**Fats**	75	46.3	87	53.7		75	41.9	104	58.1			0.41	

n, number of subjects; *, number of girls for energy intake is 176 due to missing data for physical activity; RV, reference values; significance results obtained with Pearson’s chi-square test between the percentage of boys and girls, who met or did not meet RV for daily energy and macronutrient intake, are presented with exact p-values

**Table 4:** Percentage of differently active adolescents, who met or did not meet reference value (RV) for daily energy and nutrient intake

	Boys (n = 162)					Girls (n = 176)					
	**Met** **RV**		**Did not meet RV**		**p**	**Met** **RV**		**Did not meet** **RV**		**p**	**RV** **(2016)**
	n	%	n	%		n	%	n	%		
**Energy**					0.32					0.31	boys/girls (kcal)
LPA	4	21.1	15	78.9		5	13.9	31	86.1		2300/2000
MPA	10	9.7	93	9.3		8	6.5	116	93.5		2600/2300
VPA	6	15.0	34	85		2	12.5	14	87.5		3000/2600
**Carbohydrates**					0.75					0.89	> 50% of energy from carbohydrates
LPA	12	63.2	7	36.8		28	77.8	8	22.2		
MPA	73	70.9	30	29.1		99	79.8	25	20.2		
VPA	29	72.5	11	27.5		12	75.0	4	25.0		
**Proteins**					0.07					0.55	boys / girls (g protein/day) 64 / 48 g
LPA	13	68.4	6	31.6		27	75.0	9	25.0		
MPA	84	81.6	19	18.4		82	66.1	42	33.9		
VPA	37	92.5	3	7.5		10	62.5	6	37.5		
**Fats**					0.05					0.62	30% of energy from fat
LPA	13	68.4	6	31.6		13	36.1	23	63.9		
MPA	41	39.8	62	60.2		53	42.7	71	57.3		
VPA	21	52.5	19	47.5		8	50.0	8	50.0		

n, number of subjects; LPA, less physically active; MPA, moderately physically active; VPA, vigorously physically active adolescents; significance results obtained with Pearson’s chi-square test between differently physically active adolescents are presented with exact p-values *(p < 0.008* due to multiple comparisons in 3 × 2 contingency table (Bonferroni correction))

Referring to Table 3, results demonstrated that 75% of adolescents were meeting the national recommendations for both carbohydrate and protein intakes, while only 44% and 10% of adolescents were meeting the national recommendations for fat and energy intakes, respectively. A significantly higher percentage of boys (82.7%) were meeting recommendations for protein intake than girls (67.0%) (p = 0.001). In contrast, no statistical differences in meeting the national recommendation were found between boys and girls for either energy (p = 0.18), carbohydrates (p = 0.06), or fats (p = 0.41).

Results revealed (Table 4) that there were no statistically significant differences in the percentages of adolescents who met / did not meet energy and macronutrient recommendation between differently active boys and girls.

**Fig. 1:**
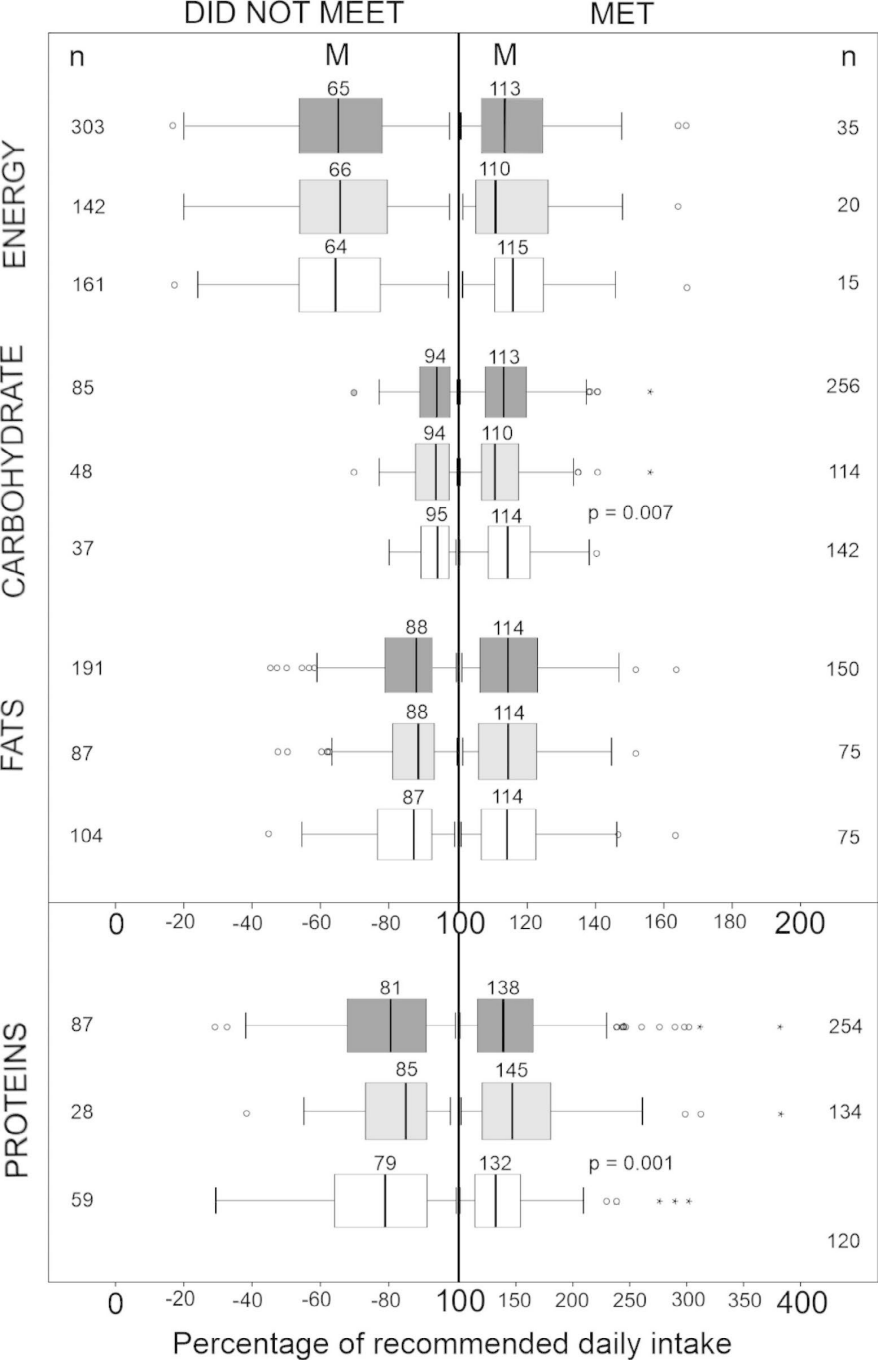
Percentage values of recommended daily energy and macronutrient intake according to the existing recommendations, presented with box-plots (results presented as minimum, maximum, sample median, as well as 1st and 3rd quartile) for all adolescents (dark gray plots), as well as separately for boys (light-gray plots) and girls (open plots) in two groups: in those who did not meet the recommendations (left side) and those who did (right side). Significant differences between boys and girls are presented with exact p-values (tested with Mann Whitney U-test). M, median; n, number of subjects

Figure 1 presents the percentage values of recommended daily intake of energy and macronutrients, for all adolescents, as well as separately for boys and girls, for the two groups: those who met the recommendations and those who did not. Results demonstrated that in the group of adolescents, who met the recommendations, girls achieved statistically higher percentages of the recommended daily intake of carbohydrates than boys (p = 0.007), while boys achieved statistically higher percentages of the recommended daily intake of proteins than girls (p = 0.001). No similar differences were observed between boys and girls with comparable extent of physical activity (results not presented).

Additional analysis between adolescents who met and did not meet the energy intake recommendations revealed that adolescents who met the recommendations had a statistically higher BMI (21.0 kg/m^2^) than adolescents who did not meet the recommendations (19.0 kg/m^2^) (p = 0.000). In addition, the extent of physical activity according to DEEPA was not significantly different between adolescents who met recommendations (8.8 kJ/kg·day) and those who did not meet the recommendations for energy intake (8.4 kJ/kg·day) (p = 0.71).

## Discussion

The results of the present study suggest that three-quarters of adolescents met the recommendations for carbohydrate (75%) and protein (75%) intake, while far lower percentage of those who met the recommendations was observed for fat (44%) and energy (10%) intake. A comparison between boys and girls demonstrated that the intake of energy and all macronutrients was significantly higher in boys than in girls in all three groups of physical activity extent, namely LPA, MPA, and VPA. In addition, protein intake recommendations were more frequently met by boys (83%) than by girls (67%), while no significant gender differences were found for other macronutrients and energy intake. The present study demonstrated that adolescent boys were significantly more physically active than girls (35% higher DEEPA median). In addition, significant differences in energy and macronutrient intake were observed in differently physically active boys, but not in differently physically active girls. Nevertheless, there were no statistically significant differences in the percentages of adolescents who met or did not meet energy and macronutrient recommendations between differently active boys and girls. However, in the group of adolescents who met the recommendations, boys achieved a higher percentage of the recommended daily protein intake, while girls consumed higher percentage of carbohydrates.

The percentage of adolescents who were meeting the recommendations for **energy** intake was very low (12% of boys; 9% of girls). In contrast, in a study by Kobe et al. [30] also conducted on Slovenian adolescents in 2012, a higher percentage of adolescents (aged 15 to 16 years) were meeting the energy intake recommendations than in our study (46% of boys and 32% of girls). Similar results to Kobe et al. [30] was reported in a study of Barić et al. [31] in Croatian adolescents (average age = 16 years) performed in 2001, where a total of 38% of adolescents were meeting the energy intake recommendations [32]. Different results between the three studies could be due to different energy assessment methods (24-h recall and the OPEN platform (Slovenia) used in our study, the 3-day weighed dietary protocol and the Prodi 5.2 Expert (Germany) in the study by Kobe et al. [30], and the food frequency questionnaire and the national food composition table (Croatia) in the study by Barić et al. [31]), and different cut-off points used for energy intake (Slovenian national recommendations adopted from the Nutrition Societies of Germany, Austria, and Switzerland [24] in our study and in the study by Kobe et al. [30], and US recommendation in the study by Barić et al. [31]).

On average, the adolescents in our sample who did not meet the national recommendations for energy intake, reached less than three quarters of recommended value for energy intake. Assuming that the reported intake is a good approximation of the actual intake, chronic energy deficiency would impair growth, development, brain function, and the endocrine system [1], as well as health-related symptoms of RED-S [19]. Nevertheless, these results should be interpreted with caution, as 24-recall method has some well-known inherent limitations, which are further discussed below.

However, it should not be excluded that the low values of energy intake and the low percentage of individuals meeting the recommendations for energy intake are also due to underreporting of energy intake. This is also supported by the fact that 75% of adolescents in our study were classified as normal weight (according to BMI). Another factor, that may contribute to the observed low percentage of adolescents who met the national recommendations for energy intake is self-assessment of the amount of physical activity [33].

A comparison of energy intake in differently physically active adolescents revealed that VPA boys had higher energy intake than LPA boys, with no other differences observed between differently active adolescents. All girls, regardless of their physical activity, had similar energy intakes. Similar to the results of the present study for girls, Ottawere et al. [34] reported no significant differences in energy intake between differently physically active adolescents of both genders. This is of particular concern because research conducted by Peklaj et al. [19] in athletes found that only 13% of athletes had no health-related symptoms of RED-S, which was particularly evident in girls.

The total **carbohydrate** intake of adolescents in our study was adequate, which was also reported in some other studies conducted in Slovenia [35], [36]. VPA boys had a statistically higher carbohydrate intake than MPA boys, while no other differences were observed between differently active adolescents. Girls with different physical activity had similar carbohydrate intakes. Ottavere et al. [34] found no significant differences in carbohydrate intake according to physical activity level in both genders. The results of Zdešar Kotnik et al. [37], obtained on the same sample as in the present study, demonstrated that the intake of salty and sweet snacks was too high in adolescents of both genders. Other studies conducted in Slovenia, have also demonstrated that adolescents consume too much sugar, snacks, and sweet drinks [26], [30], [35]. Adolescents should therefore be encouraged to choose complex and unprocessed carbohydrates such as whole grains, fresh fruits and vegetables, and low-sugar products more often.

The high percentage of adolescents (83% of boys; 79% of girls) meeting **protein** recommendations in our sample, can be explained mainly by high meat consumption [37], while other studies found a high intake of milk and dairy products among adolescents [35]. Girls consumed on average 26% less protein than boys. In addition, significantly fewer girls than boys met national recommendations for protein intake, so it is recommended that they reach for foods composed of high-quality proteins such as eggs, fish, and legumes. A comparison of adolescents with different physical activity extent demonstrated that VPA boys consumed more proteins than MPA and LPA boys, while no differences were observed for girls. Similar results were reported for both genders in the study of Otteawere et al. [34], in which more active adolescents had significantly higher protein intakes than moderately active ones. Although 80% of adolescents in the present study met recommendations for protein intake, a lower percentage of such individuals was observed in VPA and MPA girls (63% and 66%, respectively), which should therefore strive for higher protein intake.

Less than half of the adolescents in our study (46% of boys, 42% of girls) met the recommendation to obtain at least 30% of daily energy intake from fats. A similar percentage was observed in the studies of Sanchez et al. [38] (33% of adolescents) and Barić et al. [31] (22% of adolescents), while a study conducted in Australia reported a higher percentage of adolescents who met their total daily fat intake (70%) [39]. It has also been reported that in Slovenia, the adolescent population consumed the lowest amounts of fat compared to adults and elderly adults [35]. Several studies have also highlighted a worrying fact about the inadequate composition of fat intake in adolescents [35], [36], [39], [40]. It is therefore recommended that all adolescents, regardless of gender, should include more foods with unsaturated fatty acids in their daily diet and avoid foods with saturated fats.

Relative to physical activity, no significant differences in absolute fat intake were found in girls. In boys, differences were found between MPA and VPA boys, with the latter having a higher intake. Otteawere et al. [34] did not found any differences in total fat intake in both genders. Fats are a good source of energy, and to fulfill adolescent’s daily energy needs, it is necessary that they consume the right amount while considering fat composition.

In the present study, the majority of adolescents had adequate BMI (average BMI of 21.1 kg/m^2^), which would not be expected in adolescents with chronic energy deficiency. Yet, the results of Peklaj et al. [19], who conducted a study on the prevalence of RED-S-related symptoms in a sample of Slovenian competitive athletes, indicated the same issue. In addition, results demonstrated that adolescents, who did not meet the energy intake recommendations according to their reported food intake, had higher BMI than adolescent who met the recommendation according to their reported food intake. Yet, these two groups had a comparable DEEPA. These two observations indicate to an underreporting of dietary intake, particularly in individuals with higher BMI.

Finally, we would like to consider some of the potential methodological **limitations** of the present study. Although this may not be feasible in large scale studies, the use of accelerometry or heart rate monitors over self-report methods for the assessment of physical activity extent is of course preferential. Namely, self-assessment of physical activity with the questionnaire SHAPES may be subjective, leading to an overestimation of DEEPA [41], [34]. In contrast, the conservative MET values (6 MET for VPA and 3 MET for MPA) used in our calculation of DEEPA would have the opposite effect. Indeed, some guidelines recommend the use of higher MET values for VPA (7 MET) and MPA (4 MET) and a higher ratio between MET and kcal/kg·h (a ratio of more than 1 MET = 1 kcal/kg·h is recommended) [42], [43]. We therefore expect that at least some of the potential overreporting of physical activity extent has been neutralized with the use of conservative MET values in DEEPA calculation.

It is also worth considering that the recommended values for energy intake may have been too low for some individuals, because physical activity classification in our study was based on DEPPA values (i.e. estimation of average daily energy expenditure for physical activity), whereas the recommendations for energy intake are based on classifying individuals according to their physical activity level (i.e. estimation of total daily energy expenditure). However, if this were true, an even smaller percentage of adolescents than reported would meet the recommendations. Our main consideration is that the majority of subjects, who did not meet the recommendations for energy intake according to the analysis of two 24-h recalls, had an appropriate BMI. This may suggest that the adolescents were either underreporting their daily food intake or that they selected some inadequate foods in the OPEN system. However, the latter does not seem to be a plausible explanation, as data in the OPEN system are regularly harmonized with the European Food Information Resource [44]. Thus, if only feasible, we suggest that a more accurate method for the assessment of total energy intake (e.g. a weighted food diary) should be used instead of 24-h recalls. The study provides an insight into how different levels of physical activity affect energy and macronutrient intake in adolescent boys and girls. With this knowledge, it is possible to establish preventive measures to minimize the negative health consequences of inadequate nutrient and energy intake. The results of the study also reveal, which issues should be focused on (gender, especially highly active girls) while providing advice on nutrition to adolescents with different levels of physical activity.

## Conclusions and implications for research and practice

Adolescents need to ensure an adequate intake of energy and all macronutrients for normal growth and development, while maintaining regular and sufficient physical activity. Although results on energy intake obtained from 24-h recalls should be taken with caution (namely, although the majority of adolescents failed to meet recommendations for energy intake according to their reported food intake, they also had an appropriate BMI, which is an unlikely situation, if energy intake were consistently low), some important conclusions can be made. Present study demonstrated that according to their reported food intake, most Slovenian adolescents are well supplied with carbohydrates and proteins. In contrast, almost all of them failed to meet minimal recommendations for energy intake, and less than 50% met the minimal recommendations for fat intake. Particularly alarming was the energy intake data in VPA girls, who consume the same amounts of energy than MPA and LPA girls, despite being physically very active and having higher nutrient and energy needs. Therefore, the attention should be paid not only to food intake of less active, but also to that of vigorously active adolescents. To prevent negative consequences of inadequate energy and nutrient intake, knowledge about proper nutrition must be transmitted to all groups of adolescents with different activity levels, which can be obtained through various educational activities at schools or in sports clubs. Parents and coaches should also be involved, as they may influence adolescents’ eating habits.

## Data Availability

The raw data supporting the conclusions of this article will be made available by the authors upon request to gregor.starc@fsp.uni-lj.si or gregor.jurak@fsp.uni-lj.si, without undue reservation.

## Abbreviations

ACDSi: The analysis of children’s development in Slovenia
BMI: Body mass index
DEEPA: Average daily energy expenditure for physical activity
EFSA: European food safety authority
LPA: Less physically active
MET: metabolic equivalent
MPA: Moderately physically active
OPEN: Open platform for clinical nutrition
RED-S: Relative energy deficiency syndrome in sport
RV: Reference value
SHAPES: School health action, planning and evaluation system
VPA: Vigorously physically active
