# Correction: Adequacy of energy and macronutrients intake in differently active Slovenian adolescents

**DOI:** 10.1186/s40795-023-00716-x

**Published:** 2023-04-03

**Authors:** Emanuela Čerček Vilhar, Petra Golja, Gregor Starc, Barbara Koroušić Seljak, Katja Zdešar Kotnik

**Affiliations:** 1 Biotechnical Faculty, Department of Biology, University of Ljubljana, Vecna pot 111, Ljubljana, 1000 Slovenia; 2 Faculty of Sport, University of Ljubljana, Gortanova 22, Ljubljana, 1000 Slovenia; 3 Computer Systems Department, Jožef Stefan Institute, Ljubljana, 1000 Slovenia

**Correction: BMC Nutr 9, 58 (2023)**

10.1186/s40795-023-00708-x

Following publication of the original article [1], the authors identified an error in Table 4.

Tables 4 and 5th column, 6th row (% of energy intake in MPA adolescents who did not meet RV) is 90.3 and not 9.3.

The original article [1] has been corrected.
